# Comparative genome analyses reveal the unique genetic composition and selection signals underlying the phenotypic characteristics of three Chinese domestic goat breeds

**DOI:** 10.1186/s12711-019-0512-4

**Published:** 2019-11-26

**Authors:** Jiazhong Guo, Jie Zhong, Li Li, Tao Zhong, Linjie Wang, Tianzeng Song, Hongping Zhang

**Affiliations:** 1 College of Animal Science and Technology, Sichuan Agricultural University, Chengdu, 611130 China; 2 Institute of Animal Science, Tibet Academy of Agricultural and Animal Husbandry Sciences, Lhasa, 850009 China

## Abstract

**Background:**

As one of the important livestock species around the world, goats provide abundant meat, milk, and fiber to fulfill basic human needs. However, the genetic loci that underlie phenotypic variations in domestic goats are largely unknown, particularly for economically important traits. In this study, we sequenced the whole genome of 38 goats from three Chinese breeds (Chengdu Brown, Jintang Black, and Tibetan Cashmere) and downloaded the genome sequence data of 30 goats from five other breeds (four non-Chinese and one Chinese breed) and 21 Bezoar ibexes to investigate the genetic composition and selection signatures of the Chinese goat breeds after domestication.

**Results:**

Based on population structure analysis and *F*_ST_ values (average *F*_ST_ = 0.22), the genetic composition of Chengdu Brown goats differs considerably from that of Bezoar ibexes as a result of geographic isolation. Strikingly, the genes under selection that we identified in Tibetan Cashmere goats were significantly enriched in the categories hair growth and bone and nervous system development, possibly because they are involved in adaptation to high-altitude. In particular, we found a large difference in allele frequency of one novel SNP (c.-253G>A) in the 5′-UTR of *FGF5* between Cashmere goats and goat breeds with short hair. The mutation at this site introduces a start codon that results in the occurrence of a premature FGF5 protein and is likely a natural causal variant that is involved in the long hair phenotype of cashmere goats. The haplotype tagged with the AGG-allele in exon 12 of *DSG3*, which encodes a cell adhesion molecule that is expressed mainly in the skin, was almost fixed in Tibetan Cashmere goats, whereas this locus still segregates in the lowland goat breeds. The pigmentation gene *KITLG* showed a strong signature of selection in Tibetan Cashmere goats. The genes *ASIP* and *LCORL* were identified as being under positive selection in Jintang Black goats.

**Conclusions:**

After domestication, geographic isolation of some goat breeds has resulted in distinct genetic structures. Furthermore, our work highlights several positively selected genes that likely contributed to breed-related traits in domestic goats.

## Background

Modern livestock were domesticated tens of thousands of years ago [1–3], and provide an invaluable resource for fulfilling basic human needs. After dispersal throughout the world from domestication centers, domestic animals have genetically adapted to local environmental factors (e.g., temperature, humidity, and oxygen content) over time [4–7]. In addition, domestic animals have been subjected to artificial directional selection for different purposes, such as coat color and meat and milk production [7–9].

The phenotypic variation of a Mendelian trait in livestock can be determined by a single genetic locus with large effect, e.g., plumage color in Pekin ducks [10]. In such cases, artificial selection or adaptation to local conditions usually leads to a rapid increase in the frequency of the desirable allele at the population level and leaves a classical selection signature that is referred to as a “hard sweep” [11]. In another scenario, multiple independent mutations at one locus in a population introduced by e.g. migrations or mutation events are all similarly advantageous and their frequencies tend to increase slowly by selection, which produces one type of soft sweeps [11] that is usually more difficult than hard sweeps to detect using the standard methods of detection of signatures of selection [11–13]. Furthermore, artificial selection in livestock acts mainly on quantitative traits (e.g., body weight and size) and is prone to result in polygenic adaptation [14]. Based on an analysis of eight domestic cattle breeds with an ~ 800 K single nucleotide polymorphism (SNP) chip, Kemper et al. [15] reported that strong artificial selection for quantitative traits (e.g., milk yield) left little or no classic signatures of selection, whereas recent studies have revealed selection signatures for some complex traits, such as fiber and meat production traits in goats [7, 16], immune traits in sheep [16], and milk-related traits in cattle [17].

It is widely accepted that the Bezoar is the wild ancestor of domestic goats and that the domestication center was the Fertile Crescent [1, 18, 19]. After domestication, long-term selection for morphological traits created many goat breeds with diverse phenotypes (regarding e.g., coat color, horn shape, and hair type) [19], but the genetic loci that underlie these phenotypic variations in domestic goats are largely unknown, particularly for economically important traits. To address this issue, many studies have sought to identify the signatures of selection that may be associated with important traits in goats based on comparative genomic analyses. For example, Wang et al. [20] showed that the genes *FGF5* for fiber-related traits in cashmere goats, *ASIP* for coat color, and *NOXA1* for adaptation to high-altitude were under positive selection in eight goat populations. Genomic comparisons between Dazu black and Inner Mongolia Cashmere goats showed that several regions of selective sweeps were related to reproduction and production traits [21]. In a previous study, we showed that three loci involved in pigmentation (i.e., *RALY*-*EIF2S2*, *IRF4*-*EXOC2*, and *KITLG*) were under positive selection in domestic goats [22] but the evidence was weak mainly because individual genotypes were unavailable from our pooled sequence data. Compared to methods for population genetic analyses based on SNP data, those based on haplotype homozygosity and frequency could improve the power of detection for loci under selection [12, 13, 23]. Based on more than 3000 sampled goats that originated mainly from Europe and Africa, the Goat ADAPTmap consortium has recently investigated signatures of selection and the strong partitioning of diversity caused by geographic isolation [7, 24], and provided the first comprehensive picture of the global domestication and adaptation of domestic goat breeds. However, the studies of the Goat ADAPTmap consortium did not include goat breeds from China [7, 24], although large numbers of domestic goats are raised in China under different environments [19]. For example, animals that are located in the Sichuan Basin, which is surrounded by very high and extensive mountains since thousands of years ago, may have a highly distinctive genetic structure due to their geographic isolation. Moreover, the Tibetan Plateau that is adjacent to the Sichuan Basin has a very harsh environment (e.g., low temperatures and oxygen levels).

In this study, we sequenced the whole genome of 38 goats from three Chinese breeds from the Sichuan basin and the Tibetan Plateau. Then, we performed comparative population genomics analyses by comparing our sequencing data with sequence data of 30 goats from five other breeds and 21 Bezoar ibexes to investigate the evolutionary history and the signatures of selection that underlie the phenotypic differences in various goat breeds after domestication.

## Methods

### Animals and whole-genome sequencing

In this study, we sequenced the genome of 38 animals that were unrelated within three generations of three Chinese goat breeds: Jintang Black (JT) from a breeding farm in Jintang County, Chengdu Brown (CB) from a breeding farm in Dayi County, and Tibetan Cashmere (TC) from Coqen County in Tibet. Specifically, the genomic DNA of these 38 Chinese goats was extracted from whole blood samples using the E.Z.N.A. TM Blood DNA Kit (OMEGA, USA). The DNA samples of the 14 TC goats were sequenced on an Illumina HiSeq X10 sequencer in paired-end 150 bp mode at Novogene (Beijing, China), whereas the DNA samples of the 15 CB and nine JT goats were sequenced on the BGISEQ-500 platform in 2 × 100 bp mode at BGI (Shenzhen, China). We downloaded genome sequence data of 21 Bezoar ibexes (BI) and 30 other goat individuals: two Alpine (AL), two Saanen (SA), 14 Draa (from Morocco, MD), and eight Moroccan Northern (MN) goats from the Nextgen project (dataset number PRJEB3136, PRJEB5900, and PRJEB3134), and four Shaanbei Cashmere goats (SC) (PRJNA422206) from NCBI.

### Alignment and variant calling

After removing read pairs that contained adapter sequences, the raw reads were quality controlled by using the Trimmomatic software [25] (v0.36), with the following parameters: LEADING: 20, TRAILING: 20, SLIDINGWINDOW: 4:20 and MINLEN: 50 (see Additional file 1). High-quality reads were then mapped against the goat reference genome [26] (assembly ARS1, https://asia.ensembl.org/index.htm) using the ‘mem’ algorithm of BWA [27] (v0.7.12) with default parameters. We applied the Picard software (v2.10.6) (http://broadinstitute.github.io/picard/) to remove duplicated reads and then GATK (v3.8-0) [28] for local realignment around existing indels and base quality score recalibration.

For quality control of the variants, we used the GATK software to filter the raw variant calls (SNPs and indels) with the following cut-off values: QUAL < 100.0, QD < 2.0, MQ < 40.0, FS > 60.0, SOR > 3.0, MQRankSum < − 12.5, and ReadPosRankSum < − 8.0 (see Additional file 1), and VCFtools [29] to remove the variants with a minor allele frequency (MAF) lower than 0.05 and variants with more than 10% missing genotypes at the meta-population level. Finally, biallelic SNPs were extracted and used in the subsequent analyses. SnpEff [30] (v4.3) was used for SNP variant annotation and effect prediction.

### Population genetic analysis

The nucleotide diversity (π) [31] in 10-kb non-overlapping windows was first calculated using VCFtools [29]. The genome-wide linkage disequilibrium (LD) r^2^ in each breed was calculated using the PopLDdecay [32] (v3.4.0) software based on the high-quality SNPs. We also applied PLINK [33] to detect runs of homozygosity (ROH) in each goat breed, with the command ‘–homozyg-window-kb 5000 –cow –homozyg-window-snp 50 –homozyg-window-het 1 –homozyg-snp 10 –homozyg-kb 100 –homozyg-density 10 –homozyg-gap 100’. The parameter ‘–cow’ was used to indicate the number (i.e., 29) of autosomes in the goat genome since cow and goat have the same number of autosomes. Following Bertolini et al. [34], we also calculated the ROH-based inbreeding coefficient (*F*_ROH_) for each breed, which was defined as the average fraction of the genome covered by ROH, by considering a total length of 2.92 Gb for the goat reference genome (ARS1).

To explore the degree of admixture between the sampled goats, genetic admixture analysis was carried out with the ADMIXTURE [35] (v1.3.0) software by varying the number of presumed ancestral populations. We also carried out principal component analysis (PCA) implemented in GCTA [36] (v1.26.0) to analyze the genetic relationships after converting the file including biallelic SNPs into PLINK PED format with PLINK [33].

We applied the Treemix [37] (v1.13) software to infer the historical relationships and possible gene flow between populations at the population level. To identify the optimal number (“m”) of migration events, we ran 20 iterations for each migration event (m = 1–9) with a different random k value (1000–10,000) (i.e., the number of SNPs per block for the estimation of covariance matrix). We then used the OptM R package to pinpoint the optimal “m” (https://CRAN.R-project.org/package=OptM).

### Identification of selection signals and functional enrichment analysis

To identify the signals of positive selection that are driven by artificial selection and genetic adaptation to the local environment after domestication, we calculated the genome-wide fixation index (*F*_ST_, i.e. Weir and Cockerham’s estimator [38]), Tajima’s D [39] and θ_π_ ratios (i.e., θ_π_·BI/θ_π_·TC, θ_π_·BI/θ_π_·CB, and θ_π_·BI/θ_π_·JT) in 10-kb non-overlapping windows across the autosomes between domestic goat and Bezoar ibex populations using VCFtools [29]. We also calculated the haplotype homozygosity-based statistic iHH12 [40] (i.e., H12 [13]) in selscan [41], after inferring the haplotype phase and imputing the missing alleles with Beagle (v4.0) [42] with default parameters. To detect the genomic loci that are associated with adaptation to high-altitude, we also calculated the pairwise *F*_ST_ and θ_π_ ratios (θ_π_·Control/θ_π_·TC) in 10-kb windows between the highland breed (TC) and the lowland control group of breeds (CB, JT, MD, and MN). Finally, we averaged the normalized iHH12 of each biallelic SNP site in 10-kb non-overlapping windows. To reduce the number of false positives, only the outlier windows (with a number of SNPs ≥ 10) showing extremely high *F*_ST_, θ_π_ ratio, and iHH12 values (corresponding to the 5% right end of the tail) were identified as selection signals. Statistical analysis was conducted in the R Statistical Programming Language [43].

According to genome annotation, a gene was assumed to be under positive selection if it overlapped with a selection signal. To obtain an in-depth view of the biological significance of the candidate selection signals in each breed, we carried out Gene Ontology (GO), KEGG and MGI Mammalian Phenotype (MGI-MP) analyses for the identified genes using Enrichr [44]. Because no goat genome information is available in Enrichr, we used the human homolog gene symbols.

### Sanger sequencing of SNPs

Because *FGF5* has a major role in the regulation of hair fiber traits in animals [20, 45–47], we validated one novel SNP (chr6: 95418992, c.-253G>A) in the 5′UTR of *FGF5* that could result in a premature protein (for details, see the Results section). A primer pair was designed to amplify the target fragments that contained this SNP (forward primer: 5′-ACTCACTCACTCGGCATTTC-3′; reverse primer: 5′-CGTGGGAGCCATTGACTTT-3′). Similarly, given the significant role of *ASIP* in the synthesis of pheomelanin in animals [48], one SNP (chr13: 63249342, c.383G>T) in exon 3 (which is the 4th exon in NCBI) of *ASIP* was also validated using Sanger sequencing with a primer pair (forward primer: 5′-AGTGGGGCGGACGTGGATG-3′; reverse primer: 5′-GCAGGGGACTAGGCGAAGG-3′).

DNA samples of each of the five homozygous goats with either the reference or mutant allele were selected as template for PCR. All the purified PCR products were directly sequenced on an ABI 3730XL sequencer (Applied Bio-System, USA) at Tsingke Biological Biotechnology Co., Ltd. (Chengdu, China) using the forward primer. Sequences were analyzed with the DNASTAR software (v.7.1).

## Results

### Abundant genomic variation and low genetic diversity in the goat genome

**Fig. 1:**
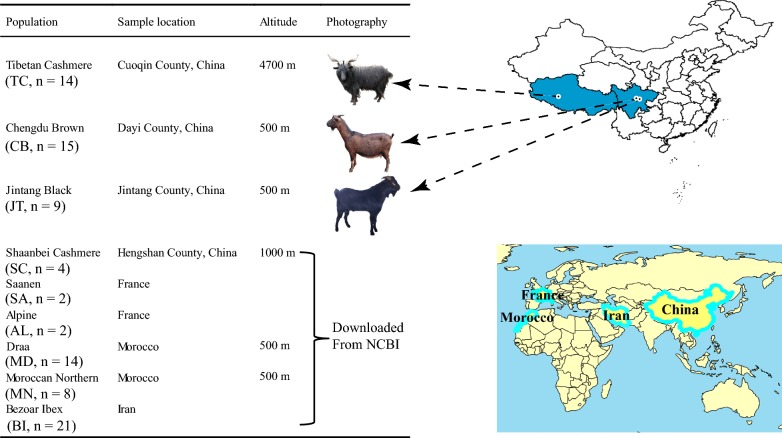
Sample information and geographic distribution of the 89 goats included in this study. Geographic locations of the eight domestic goat breeds and Bezoar ibexes included in this study: Tibetan Cashmere (n = 14), Chengdu Brown (n = 15), Jintang Black (n = 9), Shaanbei Cashmere (n = 4), Saanen (n = 2), Alpine (n = 2), Draa (n = 14), Moroccan Northern (n = 8), and Bezoar ibex (n = 21)

Based on our genome sequence data for 38 goats from three Chinese goat breeds (Fig. 1) and (see Additional file 2: Table S1) and the whole-genome sequence data from 30 individuals of five other goat breeds and 21 Bezoar ibexes downloaded from NCBI (see Additional file 3: Table S2), 19,791,420 single nucleotide variations (SNVs) (18,082,455 biallelic and 109,280 multiallelic SNPs and 1,599,685 indels) were identified across these 89 goat autosomal genomes (see Additional file 4: Table S3). At the population level (MAF ≥ 0.05), 10,656,569, 12,580,085, and 12,491,330 biallelic SNPs were detected in the CB, JT, and TC breeds, respectively (see Additional file 4: Table S3). Such a high density of SNPs (7.33 biallelic SNPs/kb across the autosomes) enabled an accurate search for signatures of selection in the goat genome. According to genome annotation, most of the SNPs (89.67%) and indels (89.13%) were located in intergenic and intronic regions, but the proportions of exonic SNPs and indels were only 0.88% and 0.26%, respectively (see Additional file 5: Table S4).

**Fig. 2:**
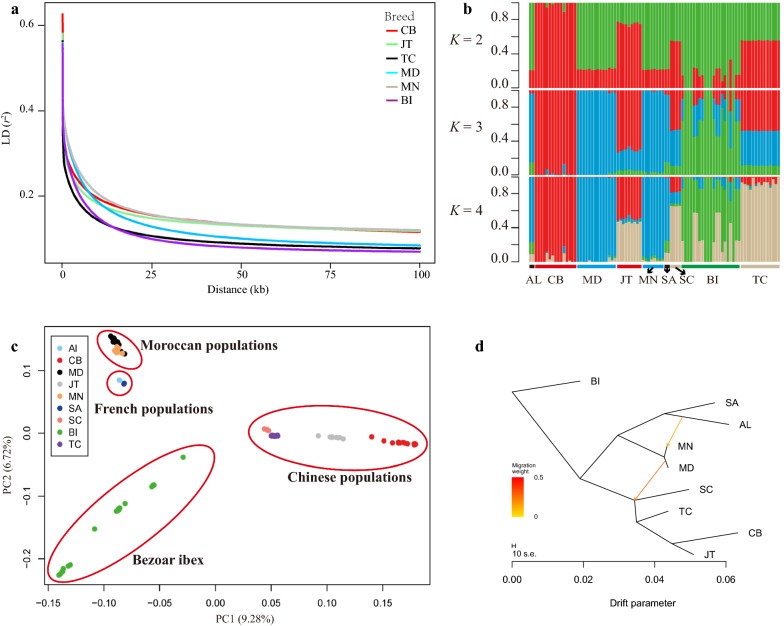
Population genetics analyses of the eight domestic goat and Bezoar ibex populations. **a** Decay of linkage disequilibrium in CB, JT, TC, MD, MN, and Bezoar ibex. **b** Proportions of genetic ancestry for the 89 sampled goats with a number of ancestral clusters ranging from 2 to 4. **c** PCA of the 89 sampled goats based on the identified biallelic SNPs. **d** Maximum likelihood tree for the eight domestic goat populations. Bezoar ibexes were chosen as the outgroup

The genome-wide average π in 10-kb non-overlapping windows for the five domestic goat (i.e., CB, JT, TC, MD, and MN) and Bezoar ibex populations ranged from 1.51 × 10^−3^ (CB) to 1.92 × 10^−3^ (MN), which indicates a low genetic diversity in each of these breeds (see Additional file 6: Figure S1a). Furthermore, the breeds with a low π had a high ROH coverage (see Additional file 6: Figures S1a, b). Among the goat breeds studied here, CB exhibited the largest number of ROH (from 1526 to 2128), the highest ROH coverage (from 407.44 to 729.21 Mb), and the highest inbreeding level (*F*_ROH_ = 0.194) (see Additional file 6: Figure S1b and Additional file 7: Table S5). Among the four categories of ROH size considered in this study, short ROH (0–250 kb) were the most frequent (ranging from 56.40% in the Bezoar ibexes to 82.23% in TC), whereas long ROH (> 1 Mb) accounted for 1.11% (TC) to 8.48% (Bezoar ibex) of the total ROH (see Additional file 6: Figure S1c). In addition, the genome-wide mean LD measured by r^2^ was largest between adjacent SNP pairs (0.56 to 0.65) in CB, JT, TC, MD, MN, and Bezoar ibex populations (Fig. 2a), and decreased rapidly to 0.2 at a distance of 5 kb. The most rapid decay of LD was observed for the Bezoar ibex followed by TC, whereas it decayed at a slightly slower rate in CB and JT from the Sichuan Basin compared to MN and MD and Bezoar ibex.

### Strong partitioning of the genetic diversity between the nine goat populations

Although the genetic admixture analysis was performed by assuming a number of ancestral populations (K) ranging from 2 to 9, the results showed that K = 4 was the most plausible number of genetically distinct clusters for the 89 sampled goats (Fig. 2b). In this scenario, all CB, TC, MN, and MD individuals represented a mixture of alleles from three populations of unknown ancestry, which do not agree with the genetic information of eight of the Bezoar ibexes. In contrast, the other 13 Bezoar ibexes were resolved as an admixture of three populations: a wild goat population, a Moroccan population, and a Tibetan goat population. The JT and SC individuals from China showed admixture of the ancestral populations, to some extent. According to the genome-wide *F*_ST_ in 10-kb sliding windows, CB was the most diverged breed (average weighted *F*_ST_ = 0.22) from the Bezoar ibex, followed by JT (*F*_ST_ = 0.17), TC (*F*_ST_ = 0.14), MD (*F*_ST_ = 0.14), and MN (*F*_ST_ = 0.14) (*P* < 2.2 × 10^−16^, Mann–Whitney U test). In addition, a moderate divergence was observed between CB and JT (*F*_ST_ = 0.11) or TC (*F*_ST_ = 0.15).

PCA clearly showed that all the individuals of each domestic goat breed clustered close together in small groups (Fig. 2c), whereas the 21 Bezoar ibexes clustered loosely, in accordance with the results of the genetic admixture analysis with K = 3 and 4. Strikingly, the first eigenvector separated the four Chinese goat populations (CB, JT, SC, and TC) from the Bezoar ibexes (9.28% of the explained variance), whereas the two Moroccan (MD and MN) and the two European breeds (SA and AL) were further separated from the Bezoar ibexes along the second eigenvector axis (6.72% of the explained variance). According to the analysis without considering migration events, the eight domestic goat breeds were divided into two large clusters at the population level: one that comprised the European and Moroccan populations and another that included the Chinese populations, which was consistent with the PCA and the population coancestry analysis of individual genomes (see Additional file 8: Figure S2a). When migration edges (m = 1–9) were added to the maximum likelihood (ML) tree, the optimal number of migration events was found to be 2 using OptM (see Additional file 8: Figure S2b and 2c). In this scenario, two migration edges were observed from AL (France) to MN (Morocco) and from MD (Morocco) to SC (China) (Fig. 2d).

### Positive selection signals in the three Chinese goat breeds

**Fig. 3:**
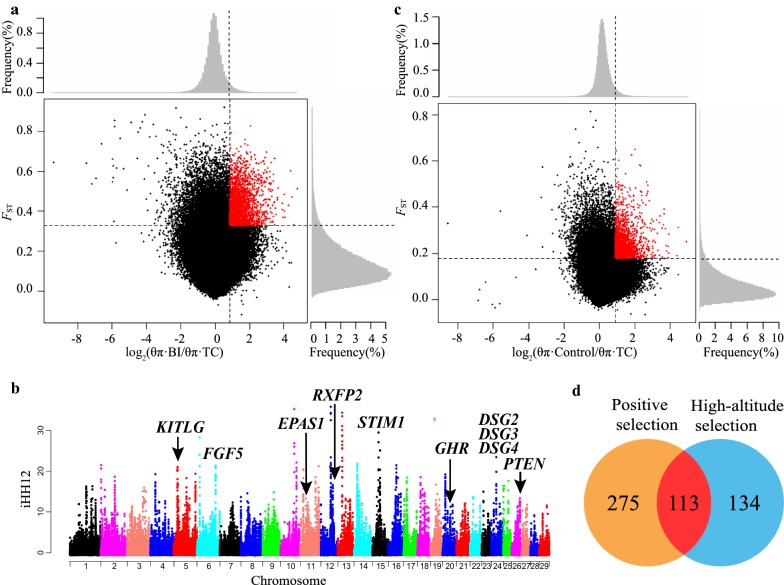
Genome-wide selection signals identified in Tibetan Cashmere goats. **a** Distribution of log_2_(θ_π_ ratios) and *F*_ST_ values calculated in 10-kb sliding windows between TC and Bezoar ibex. The data points in red (corresponding to the top 5% of the empirical distribution) indicate the signatures of selection in TC. **b** Manhattan plot of iHH12 across all 29 autosomes with different colors in TC. The iHH12 values were calculated in 10-kb sliding windows. **c** Distribution of log_2_(θ_π_ ratios) and *F*_ST_ values calculated in 10-kb sliding windows between TC and the lowland group of goat breeds (including CB, JT, MD, and MN). **d** Venn diagram for the positively selected genes identified in TC from two different comparisons (i.e., TC versus Bezoar ibex and TC versus the four lowland breeds (CB, JT, MD, and MN)

To identify signatures of positive selection, we calculated iHH12 scores and pairwise *F*_ST_ and θ_π_ ratios in 10-kb sliding windows across the autosomes between the three Chinese goat breeds and the Bezoar ibexes. In total, 1089, 749, and 664 outlier windows (corresponding to 0.44, 0.30, and 0.27% of the genome) were identified as regions of selective sweeps in TC (iHH12 > 1.93, *F*_ST_ > 0.33, and θ_π_ ratio > 1.81), CB (iHH12 > 2.33, *F*_ST_ > 0.46, and θ_π_ ratio > 2.89), and JT (iHH12 > 2.04, *F*_ST_ > 0.38, and θ_π_ ratio > 2.18), respectively (Fig. 3a, b) and (see Additional file 9: Figures S3a–d and Additional file 10: Table S6). Based on genome annotation, we identified 388, 248, and 245 genes under selection (Ensembl ID) in the TC, CB, and JT goats, respectively (see Additional file 11: Table S7 and Additional file 9: Figure S3e). Although the CB and JT breeds are present in neighboring regions in the Sichuan basin, only 28 genes under selection were shared between these breeds (see Additional file 9: Figure S3e), which indicates that their respective selection targets differed. In addition, we detected 322, 206, and 181 breed-specific genes under selection in the three Chinese breeds, respectively (see Additional file 9: Figure S3e).

To better understand the biological implications of the selection signals, a functional enrichment analysis was conducted for the positively selected genes (PSG) in each breed (see Additional file 12: Table S8). At the molecular and phenotypic levels, no significantly enriched terms (*P*_adj_ > 0.05) were found for the genes under selection in CB. In contrast, the PSG in JT were significantly enriched in five GO terms, i.e. ‘Molecular Functions’ (*P*_adj_ < 0.05), including adenyl ribonucleotide binding (11 PSG, e.g., *POPDC3*, *ACTC1* and *CDK6*), phosphatidylinositol-4,5-bisphosphate 3-kinase activity (5 PSG, e.g., *PDGFRB*, *FGF9* and *KIT*) and ATP binding (9 PSG, e.g., *ACTC1*, *CDK6* and *TGFBR1*). Although the enrichment analysis did not provide strong evidence of links between the selective sweep regions and economically important traits, several PSG may be relevant for the CB breed (e.g., *POU1F1*, *GHRH* and *GABRA2*) and the JT breed (e.g., *ASIP*, *LCORL*, *MITF* and *KIT*) (Table 1), based on their biological functions and the phenotypic characteristics of the two breeds.

**Table 1:** Summary of several positively selected genes detected in Chengdu Brown and Jintang Black goats

Breeds	Sweep region (Mb)	Gene	iHH12	*F*_ST_	π ratio	Traits	Molecular function
CB	chr6: 34.24-34.25	*POU1F1*	3.43	0.51	5.78	Litter size and growth	Activates growth hormone and prolactin genes
CB	chr6: 65.41-65.45	*GABRA2*	6.27	0.61	3.66	Behavior	Mediates neuronal inhibition by binding to the GABA/benzodiazepine receptor and opening an integral chloride channel
CB	chr13: 65.76-65.77	*GHRH*	2.38	0.57	3.19	Growth	Stimulates the secretion of growth hormone
JT	chr6: 37.97-38.06	*LCORL*	13.79	0.47	3.69	Body size	DNA-binding transcription factor activity
JT	chr6: 70.73-70.79	*KIT*	13.39	0.68	5.10	Coat color	Plays roles in cell survival and proliferation, hematopoiesis, stem cell maintenance, gametogenesis, mast cell development, migration and function, and in melanogenesis
JT	chr13: 63.24-63.25	*ASIP*	2.19	0.52	3.15	Coat color	Regulates melanogenesis. The binding of ASIP to MC1R precludes alpha-MSH initiated signaling and thus blocks production of cAMP, leading to a down-regulation of eumelanin (brown/black pigment) and thus increasing synthesis of pheomelanin (yellow/red pigment)
JT	chr22: 31.52-31.76	*MITF*	5.77	0.47	3.39	Coat color	Regulates the expression of genes with essential roles in cell differentiation, proliferation and survival

At the phenotypic level, the PSG detected in TC were significantly enriched in 12 MGI-MP terms (*P*_adj_ < 0.05) (see Additional file 12: Table S8). Seven of these categories were directly involved in circulation development and related diseases, including trabecula carnea hypoplasia (6 PSG, e.g., *ANKRD17*, *TGFBR3* and *LATS2*), hemorrhage (13 PSG, e.g., *PLVAP, MGRN1* and *EPAS1*), thin myocardium (7 PSG, e.g., *ANKRD17*, *TGFBR3* and *WNK1*), and cyanosis (11 PSG, e.g., *YY1*, *NDST1* and *NBEA*). In addition, two enriched terms were also related to immunity: decreased thymocyte number (8 PSG e.g., *KITLG*, *IL7* and *PRKDC*) and abnormal splenic cell ratio (5 PSG, e.g., *SELL*, *NCAPH2* and *REL*).

### Many signatures of selection underlie adaptation to high-altitude or cashmere traits in Tibetan Cashmere goats

**Fig. 4:**
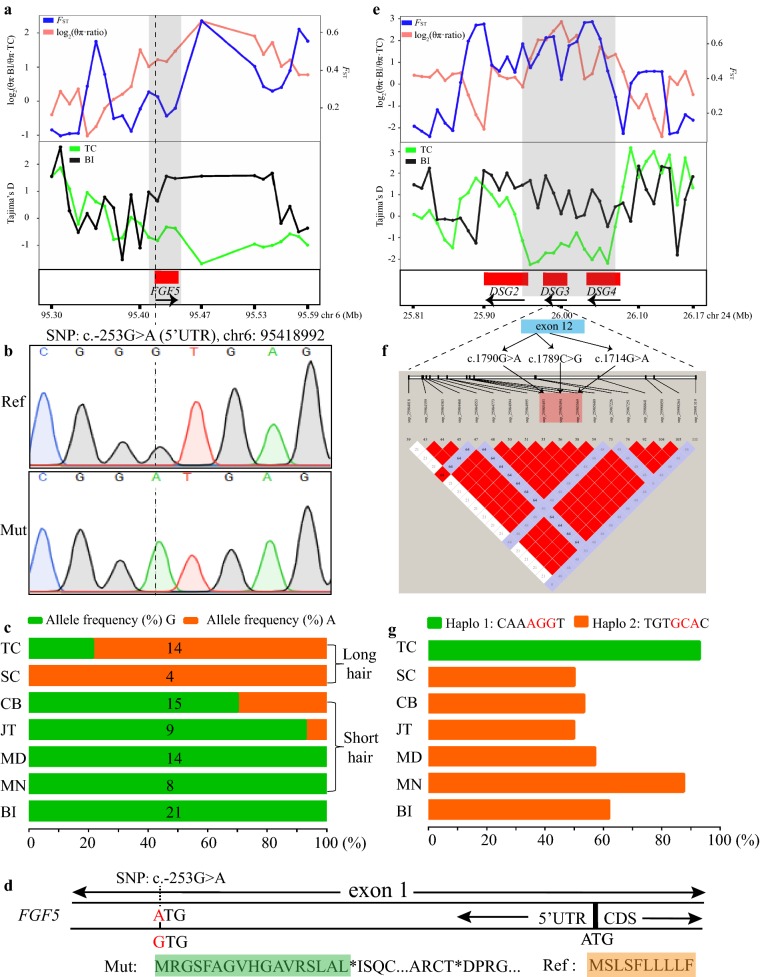
Characterization of two selection signals encompassing the genes related to hair growth in TC. **a** Log_2_(θ_π_ ratios), *F*_ST_, and Tajima’s D values in 10-kb outlier windows overlapping with the *FGF5* gene. **b** Representative Sanger sequence traces of two alleles at the c.-253G > A SNP site in the 5′-UTR of *FGF5*. **c** The allele frequency at the c.-253G > A SNP site in the 5′-UTR of *FGF5* in five domestic goat breeds (CB, JT, TC, MD, and MN) and Bezoar ibexes. **d** The predicted premature amino acid sequence caused by the mutant allele ‘*A*’ at the c.-253G > A SNP site in the 5′-UTR of *FGF5*. **e** Log_2_(θ_π_ ratios), *F*_ST_, and Tajima’s D values in 10-kb outlier windows encompassing *DSG2*, *DSG3*, and *DSG4*. **f** Pairwise LD between the three missense SNPs (chr24: 25,985,493, 25,985,494, and 25,985,569) and other SNPs in the *DSG3* gene in TC. Squares in pink or red indicate significant LD between SNP pairs (bright red indicates pairwise D′ = 1). **g** The haplotype frequencies composed of the seven SNPs showing perfect LD in the five domestic goat breeds (CB, JT, TC, MD, and MN) and Bezoar ibexes

To investigate the genetic loci that underlie adaptation to high-altitude in the domestic goat genome, we conducted a pairwise comparison of the genome-wide variations between the highland breed (i.e., TC) and the four lowland breeds (i.e., CB, JT, MD, and MN) as the control group. It is noteworthy that the four lowland goat breeds have short hair. Thus, the selection signals associated with hair-related traits can be included here. In total, 731 outlier windows (iHH12 > 1.93, *F*_ST_ > 0.18, and θ_π_ ratio > 1.85) (Fig. 3c) and (see Additional file 13: Table S9) that encompassed 247 genes (Fig. 3d) and (see Additional file 14: Table S10) were detected as selection signals in TC. Among these genes, 113 (e.g., *EPAS1*, *KITLG*, *DSG3*, *RXFP2* and *SOX6*) overlapped with the positively selected genes identified in the comparison between TC and Bezoar ibexes (Fig. 3d), which suggests that they may be involved in adaptation to high-altitude or cashmere traits. The MGI-MP analysis showed that these genes under selection were significantly (*P*_adj_ < 0.05) enriched in pigmentation, hair growth, and bone and nervous system disorders (see Additional file 15: Table S11), including diluted coat color (e.g., *FIG4*, *SPAG9* and *MKLN1*), abnormal hair follicle morphology (6 PSG, e.g., *FIG4*, *TRPS1* and *DSG3*), abnormal skeleton development (7 PSG, e.g., *TGFBR3*, *KITLG* and *THRB*), abnormal chondrocyte morphology (6 PSG, e.g., *CHUK*, *IDUA* and *TRPS1*), decreased compact bone thickness (6 PSG, e.g., *NCOR2*, *FIG4* and *KITLG*), abnormal telencephalon morphology (5 PSG, e.g., *NCOR2*, *NDST1* and *LHX2*), and abnormal synaptic depression (2 PSG, *PTEN* and *MAPT*). We also found that three other positively selected genes, *FGF5*, *DSG3*, and *DSG4*, were significantly enriched in hair growth (*P* < 0.05), as described below, considering that hair growth or normal skin function is very important for adaptation to low temperatures or strong UV radiation.

### Two selective sweep regions related to hair growth in Tibetan Cashmere goats

Among the signatures of selection associated with adaptation to high-altitude, a signature between 95.41 and 95.45 Mb on chromosome 6 (iHH12 = 12.32, *F*_ST_ = 0.47, and θ_π_ ratio = 3.34) was detected in TC (Fig. 3b, c), which was also supported by the Tajima’s D values (Fig. 4a). One hundred and eighty SNPs and 18 indels were detected across the nine populations analyzed (see Additional file 16: Table S12), including three synonymous substitutions in the exons (c.273T>C, c.450A>G, and c.699T>A) of *FGF5*, which is a key regulator of hair length.

The absence of functional changes in the predicted FGF5 amino acid sequence prompted us to examine the effect of SNPs in noncoding regions. In the 89 goat genomes analyzed, only two SNPs (chr6: 95,418,992, 95,419,200; c.-253G>A and c.-45G>A) were detected in the 5′-UTR of *FGF5* (see Additional file 16: Table S12), and the first mutation (c.-253G>A) was validated by Sanger sequencing (Fig. 4b). These two SNPs displayed a high level of divergence (*F*_ST_ = 0.66 and 0.41, respectively) between TC and the other goat breeds (see Additional file 16: Table S12). The major allele at the c.-253G>A SNP in TC is the mutant allele “*A*”, with a frequency of 78.57%, whereas in the other populations it is the reference allele, “*G*” (JT: 92.86%, CB: 70.00%, MD: 100.00%, MN: 100.00%, and BI: 100.00%) (Fig. 4c). In addition, all four SC individuals were homozygous *AA*. As shown in Fig. 4d, the mutant allele “*A*” introduces a start codon that results in a premature protein with an amino acid sequence that is completely different from the normal amino acid FGF5 sequence.

Compared to the Bezoar ibexes and the four lowland domestic goat breeds, the genomic region that harbors exon 1 of *DSG2* and *DSG3*, and exons 2 to 15 of *DSG4*, at 25.95–26.07 Mb on chromosome 24, exhibited strong evidence of positive selection (iHH12 = 23.46, *F*_ST_ = 0.65, and θ_π_ ratio = 6.25) in TC (Figs. 3a–c, and 4e) and (see Additional file 7: Table S5 and Additional file 10: Table S6). In total, 865 SNPs and 89 indels were detected across the nine populations analyzed (see Additional file 16: Table S12), including six and seven missense SNPs in the exons of *DSG3* and *DSG4*, respectively. Moreover, the pairwise *F*_ST_ for each biallelic SNP in this region showed that 136 SNPs were highly divergent (average *F*_ST_ > 0.80) (see Additional file 16: Table S12) between TC, the four lowland goat breeds (CB, JT, MD, and MN) and the Bezoar ibexes, and included three missense SNPs in exon 12 of *DSG3* (c.1790G>A, c.1789C>G, c.1714G>A; chr24: 25,985,493, 25,985,494, and 25,985,569; average *F*_ST_ = 0.91, 0.91, and 0.83, respectively). Strikingly, the first of these two single nucleotide substitutions were adjacent and resulted in a change in arginine to glutamic acid (Arg597Glu), and the third mutation caused a glycine to serine (Gly572Ser) substitution. The estimated pairwise r^2^ showed substantial LD (r^2^ > 0.2) between the SNPs located in the region 25,981,644–25,990,264 bp in TC (Fig. 4f). In particular, perfect LD (D′ = 1 and r^2^ = 1) was observed between the above three missense SNPs, three other SNPs in intron 12 (c.1891+ 619T>C, c.1891+ 398G>A, and c.1891+ 397T>A; chr24: 25,984,773, 25,984,994, and 25,984,995) and one SNP in intron 11 (c.1640-37T>C; chr24: 25,985,680). Accordingly, only two haplotypes were detected in TC, Haplo1 (CAAAGGT) and Haplo2 (TGTGCAC). The three missense sites (i.e., c.1790G>A, c.1789C>G, and c.1714G>A) tagged the two haplotypes, with the AGG- allele tagging Haplo1 and the GCA- allele tagging Haplo2 (Fig. 4g). In TC, Haplo1 was almost fixed, with a frequency of 92.9%, but none of the individuals were homozygous for Haplo2. However, Haplo2 was present in a majority of the Bezoar ibexes (61.9%) and the lowland goat populations (CB: 53.3%, JT: 49.9%, MD: 57.1%, and MN: 87.5%) (Fig. 4g).

### Several selection signals overlap with pigmentation genes in the three Chinese goat breeds

**Fig. 5:**
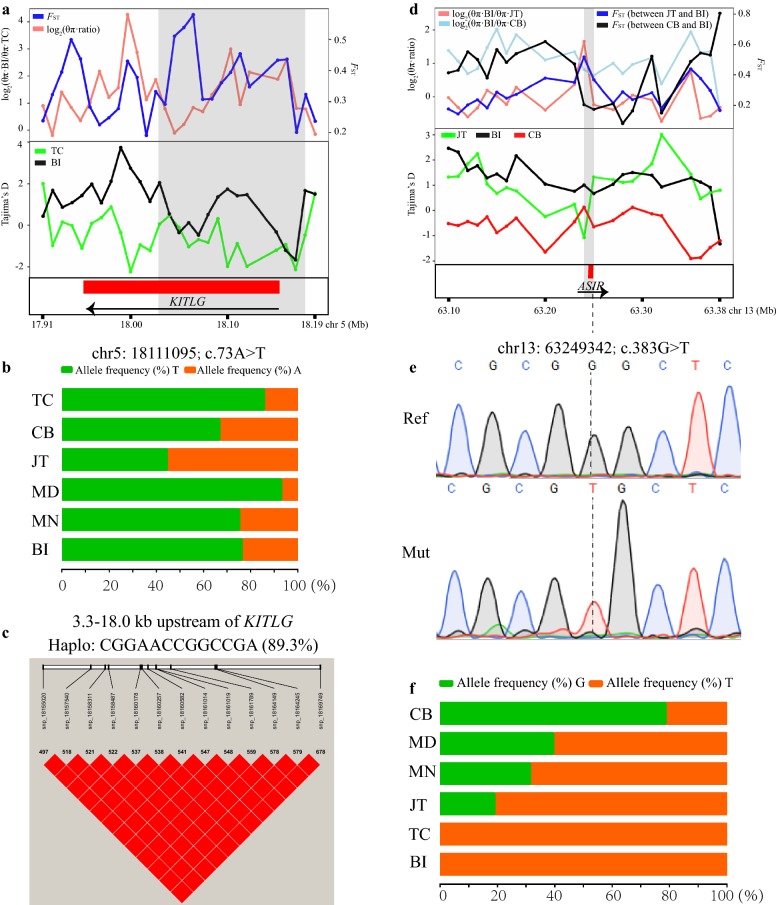
Characterization of two selection signals encompassing the pigmentation genes. **a** Log_2_(θ_π_ ratios), *F*_ST_, and Tajima’s D values in 10-kb outlier windows overlapping with *KITLG*. **b** The allele frequency of the c.73A > T SNP within *KITLG* in five domestic goat breeds (CB, JT, TC, MD, and MN) and Bezoar ibexes. **c** The high pairwise LD between the 13 SNPs upstream of *KITLG* in TC. **d** Log_2_(θ_π_ ratios), *F*_ST_, and Tajima’s D values in 10-kb outlier windows encompassing *ASIP*. **e** Representative Sanger sequence traces of two alleles at the c.383G > T SNP site within *ASIP*. **f** The allele frequency of the c.383G > T SNP within *ASIP* in the five domestic goat breeds (CB, JT, TC, MD, and MN) and Bezoar ibexes

In TC, a selective sweep region (18.02–18.18 Mb) that overlapped the *KITLG* on chromosome 5 was assumed to be under positive selection (Figs. 3a, b, and 5a) and (see Additional file 10: Table S6, and Additional file 11: Table S7) and was associated with adaptation to high-altitude (Fig. 3c) and (see Additional file 13: Table S9 and Additional file 14: Table S10). In particular, among the JT, CB, TC, MD, and MN and Bezoar ibex populations, the π values for this chromosomal region were lowest in TC (see Additional file 17: Figure S4). Among the 1785 SNPs and 164 indels detected in the nine goat breeds analyzed here (see Additional file 16: Table S12), one missense SNP was observed in exon 2 of the *KITLG* gene (c.73A>T, p.Thr25Ser), but no significant differences in allele frequency were found between TC and the other goat populations. Furthermore, the corresponding small region between 18.10 and 18.17 Mb had a very low Tajima’s D value. In this region (Fig. 5b), 13 SNPs exhibited perfect pairwise LD (D′ = 1 and r^2^ = 1), and the dominant haplotype was ‘CGGAACCGGCCGA’, with a high frequency of 89.3%, whereas these sites segregated in the Bezoar ibex and other domestic populations (i.e., CB, JT, TC, MD and MN) (Fig. 5c).

In this study, three pigmentation genes were assumed to be under selection in JT, including *ASIP* at 63.24–63.25 Mb on chromosome 13 (iHH12 = 2.19, *F*_ST_ = 0.52, and θ_π_ ratio = 3.15), *MITF* at 31.52–31.76 Mb on chromosome 22 (iHH12 = 5.77, *F*_ST_ = 0.47, and θ_π_ ratio = 3.39), and *KIT* at 70.73–70.79 Mb on chromosome 6 (iHH12 = 13.39, *F*_ST_ = 0.68, and θ_π_ ratio = 5.10) (Table 1 and Fig. 5d) and (see Additional file 7: Table S5). However, *ASIP* is a relevant gene due to its important functions in the regulation of the synthesis of eumelanin and pheomelanin and the uniform black coat color of JT (Fig. 1). It should be noted that although this region was not assumed to be under selection in CB (with a black-and-tan coat color) based on the outlier approach, the region that overlapped *ASIP* showed a relatively high level of divergence (*F*_ST_ = 0.20) and low nucleotide diversity (θ_π_ ratio = 1.75) between CB and Bezoar ibexes (Fig. 5d). Among the 13 SNPs and three indels that were detected in *ASIP* (see Additional file 16: Table S12), one SNP (c.383G>T) caused a Gly-Val mutation (p.Gly128Val) in exon 3 of *ASIP*, which was validated by Sanger sequencing (Fig. 5e). However, JT and the other goat breeds carried mainly the mutant allele “*T*” at this site, whereas the major allele in CB was the same as the reference allele “*G*”. The frequencies of the reference allele “*G*” were 78.57, 39.29, 31.25, 18.75, 0.00, and 0.00 in CB, MD, MN, JT, TC, and Bezoar ibex, respectively (Fig. 5f).

## Discussion

### Genetic composition and relationships between the nine goat populations analyzed

In this study, we investigated the genetic composition and relationships and the signatures of selection after domestication in three native Chinese goat populations based on the genome sequence data of 68 domestic goats and 21 Bezoar ibexes. The population-based metrics (e.g., π, LD, and ROH) within a breed reflect evolutionary factors such as natural/artificial selection, demographic history, and migration. For example, the genome-wide nucleotide diversity in zebu (i.e., a primitive *Bos indicus* breed) is much higher than that in commercial cattle breeds (approximately 1.4 × 10^−3^), including Angus and Holstein cattle [6, 49]. Genetic diversity across a variety of goat populations based on π values has been reported [50–52]. However, the higher mutation rates and the small number of loci in the mitochondrial genome make it difficult to compare these results with those of our study. Compared to whole-genome studies in native cattle [6, 49] and sheep [5, 53], the genome-wide nucleotide diversity in the goat genomes was lower, which is consistent with a previous report [54]. Furthermore, the genome-wide LD decay rates in the three Chinese goat breeds were very similar to those in other native goat breeds [55, 56] but slightly higher than those in Boer, Nubian, and Toggenburg goats [55].

ROH refer to genomic segments that show consecutive homozygous genotypes and have been used to measure inbreeding in livestock [57], including domestic goats [34, 58, 59]. At the genome-wide level, the total ROH length in the three Chinese goat breeds was similar to that in other goat breeds [34, 58, 59]. As shown in previous work [34], the increased homozygosity in local goat breeds is a consequence of the small size of the populations, inbreeding, and even geographic isolation. Among the goat breeds included here, the largest ROH number and genome coverage were observed in CB, an ancient native breed, which implies a small population size with inbreeding.

A recent study demonstrated the strong partitioning of the genetic diversity in domestic goats around the world, which is caused by geographic isolation or decreased local gene flow driven by humans [24]. Based on the population genetic analysis, we found that TC and CB goats inherited ancestral genetic information that differed considerably from that of the Bezoar ibexes. Furthermore, CB goats are significantly more divergent from the Bezoar ibex than the Bezoar ibex from the other domestic goat populations. Therefore, these results collectively suggest that the genetic composition of this breed is highly distinctive and is very likely caused by isolation due to geographical barriers (i.e., mountains), which is supported by previous findings on other species from the Sichuan Basin [60, 61]. A striking finding in our study is that the genomes of some Bezoar ibexes were found to result from an admixture of three ancestral populations: a wild goat population, a Moroccan population, and a Tibetan goat population, which indicates that the Bezoar ibex has hybridized with domesticated breeds showing close genetic relationships to TC and Moroccan goats.

### Positively selected genes related to adaptation to high-altitude

In the past several years, there has been a growing interest for the identification of genetic loci that contribute to adaptation to high-altitude in humans [62, 63] and animals [4, 64, 65]. Compared to the genome of Bezoar ibexes, we identified many positively selected genes in TC. Interestingly, 20 of these positively selected genes (e.g., *EPAS1*, *PTEN*, *RXFP2*, *SOX6* and *RFX4*) were also detected in Tibetan sheep, suggesting common targets of selection since both species have genetically adapted to high altitude [53]. Furthermore, the comparison to lowland domestic goats showed that the diversifying selected genes that underlie adaptation to high-altitude were involved mainly in abnormal bone, nervous system and hair follicle development in TC, which is consistent with the phenotypic characterization of this breed (e.g., small body size and cashmere traits).

Previous studies have revealed the biological functions of some of these selected genes in biological processes, which support our findings. For example, the *RXFP2* gene is not only related to testicular dysplasia in humans and mice [66, 67] but also plays important roles in bone metabolism and osteoporosis [68]. In wild sheep, the *RXFP2* locus was identified as a signature of selection underlying sexual selection for female choice [69, 70] due to its significant functions in bone development. Several SNPs and an insertion within or around *RXFP2* are significantly correlated with horn types in domestic sheep [71–73]. Recently, Pan et al. verified that *RXFP2* was under strong selection associated with unique horn phenotypes (i.e., greater horn length and spiral and horizontally extended horn shape) as a response to semi-feralization in Tibetan sheep [53]. Furthermore, a signature of selection overlapping with *RXFP2* was identified in goats from Southwestern Europe and central Asia [7], particularly in Thari and Blanca de Rasquera goats. Although two missense SNPs were found in the nine goat populations examined in this study, the differences in allele frequency did not support the assumption that they may be under selection.

It has been established that *EPAS1*, which encodes HIF-2α, is involved in complex oxygen sensing and regulates the expression of many genes [74]. *PTEN* is a tumor suppressor gene with phosphatase activity, and loss of this gene facilitates HIF-1α-mediated gene expression [75]. Recent studies demonstrated that *EPAS1* and *PTEN* were under positive selection in native Tibetan humans [62, 64, 76], dogs [4, 64] and goats [77] driven by adaptation to high-altitude. In addition to the essential roles of cartilage formation [78], a genome-wide significant association between one SNP in *SOX6* and blood pressure traits has been reported in humans [79]. In the following sections, we discuss mainly about several genes under selection that were highlighted and could be promising candidate genes underlying hair growth or coat color in goats.

### The signature of selection encompassing the *FGF5* gene

Cashmere-related traits (e.g., fiber length) are the most important economic traits in TC and are shaped by artificial selection and adaptation to low temperatures (the average yearly temperature is below 0 °C in Coqen county). *FGF5* is a key regulator of hair fiber traits in animals [20, 45, 46], and its disruption via the CRISPR/Cas9 system leads to longer fibers in genome-edited goats [47, 80] and sheep [81]. However, the natural causal mutations underlying these phenotypes in cashmere goats are still unknown.

In this study, we found that one SNP (c.-253G>A) in the 5′-UTR of *FGF5* resulted in a start codon that could lead to a premature/dysfunctional protein. Importantly, very large differences in allele frequency at this site were observed between cashmere goats (i.e., TC and SC) and the goat populations with short hair. Moreover, all four SC individuals were homozygous for the mutant allele. Therefore, this mutation is likely a causal variant underlying a cashmere-related trait in TC and is driven by artificial selection and adaptation to low temperatures.

### The strong signature of selection including three *DSG* family genes

In addition to the well-known genes under selection discussed above, the genomic loci that encompass *DSG2*, *DSG3*, and *DGS4* showed strong evidence of a selective sweep in the TC goats analyzed here. A recent exome sequencing study demonstrated that *DSG3* was the most divergent locus between the Tibetan goat populations from Bange and Ritu and the lowland populations [77]. As an important class of the cadherin supergene family, desmogleins include four members (i.e., DSG1, DSG2, DSG3, and DGS4) that mainly function as cell adhesion molecules for keratinocyte adhesion in skin [82, 83]. DSG could serve as targets for treating skin diseases and are involved in the autoimmune function in humans [84]. For example, mutations in *DSG1* can cause the striate palmoplantar keratoderma skin disease in humans [85]; a homozygous deletion in *DSG3* leads to the loss of keratinocyte adhesion and a blistering phenotype in mice [86]. In addition, DSG1 aids in the recovery of epidermal differentiation after acute UV light exposure [87]. Our previous study revealed a significant difference in the abundance of DSG3 protein between guard fiber and fine fiber in SC goats [88], implying its important role in hair.

Together with different population statistics, the core selected site seems to encompass the three missense SNPs in exon 12 of *DSG3* in TC, which was validated in ten Chinese goat populations by Sanger sequencing [89]. In addition, four other SNPs showed perfect LD with the three missense mutations, and accordingly, only two haplotypes were detected in TC: i.e. Haplo1 (CAAAGGT), which is almost fixed, and Haplo2 (TGTGCAC) for which there were no homozygous individuals. These results are consistent with a previous study [89]. In contrast, these SNP sites continue to segregate in the Bezoar ibex and other domestic goat populations. Notably, the same previous study [89] did not support the assumption that *DSG3* was functionally directly related to a cashmere-related trait, because distinct haplotypes were found between TC goats and lowland cashmere goats. In summary, the accumulation of these missense variants in TC is due to their beneficial effects on the function of *DSG3* in skin and hair, which further contributed to their adaptation to high-altitude.

### Genes under selection related to coat color

In this study, because of the phenotypic variations in coat color in the nine goat populations analyzed, we examined whether some of the pigmentation genes were under selection. First, the genomic region overlapping the *KITLG* gene was identified as a signature of selection in TC, which is consistent with our previous findings based on pooled sequencing [22]. The comparison of nucleotide diversity values in domestic goats from Iran and Morocco revealed that similar selection signals were present in the Bezoar ibexes [16]. Based on the π values for the region around *KITLG* observed in our study for the Bezoar ibex and the five TC, CB, JT, MD, and MN breeds, the result reported by Alberto et al. [16] may be a false positive due to sampling bias. Furthermore, whole-genome resequencing studies have revealed that similar regions are under strong selection in domestic sheep [16, 90] and Berkshire pigs [91].

Previous studies have demonstrated the significant roles of *KITLG* in the regulation of skin and hair pigmentation, and one mutation in the regulatory region of *KITLG* was shown to be a causal variant controlling blond hair in humans [92, 93]. *KITLG* is more strongly correlated with adaptation to low temperatures (i.e., thermogenesis) than UV radiation in Asian populations [94]. In livestock, *KITLG* is significantly associated with UV-protective eye area pigmentation in cattle [95] and reproductive traits in goats [96] and horses [97]. Although there was one missense SNP in the coding region of *KITLG*, the moderate difference in allele frequency between the goat populations did not support this mutation as a causal variant. Strikingly, the SNPs that are in strong LD in the upstream region of *KITLG* show a large difference in allele frequency between TC and the other goat breeds, which indicates that the causal variants may be located in regulatory regions, in accordance with previous findings in goats [16], sheep [16, 90], and humans [92]. However, we were unable to speculate about the causal mutations due to the lack of molecular experiments. Considering the extreme environmental conditions (e.g., strong UV radiation and low temperatures) on the Tibetan Plateau, we concluded that the genomic region encompassing *KITLG* was under strong selection in TC, mainly due to its pleiotropic effects.

Three pigmentation genes, *ASIP*, *KIT*, and *MITF*, were assumed to be under selection in JT, and we examined more closely *ASIP* because of its major role in the regulation of the synthesis of eumelanin and pheomelanin in animals [48]. In our study, we detected a selection signal that completely encompassed *ASIP* in JT, which was in accordance with the findings in Taihang Black goats [20]. Although *ASIP* was not under selection in CB, it has been reported that the region surrounding this gene is in a signature of selection in Alpine, Poitevine, Valdostana [7], and Nanjiang Yellow [22] goats with a black-and-tan pigmentation phenotype. More importantly, missense SNPs in *ASIP* and copy number variations in goats have been reported [98, 99].

We identified a missense mutation (c.383G>T; p.Gly128Val) in exon 3 of *ASIP* that was also found in European goat breeds from Italy [100], but the patterns of allele frequency at this site were similar in all the studied goat populations except for CB in our study. In contrast, its allele frequency in CB was close to that (0.84) in Murciano-Granadina goats, which is consistent with the coat color patterns of both breeds [100]. However, this SNP did not show a significant association with coat color in Murciano-Granadina goats [100]. Furthermore, we did not detect a selection signal overlapping with *MC1R* in domestic goats, although the SNPs in the regions upstream and downstream of *MC1R* could separate black and white goats [7]. Based on a candidate gene association analysis, none of these SNPs have been significantly associated to red or yellow coat color, to date [101–103]. In summary, the genetic loci that underlie black/red or yellow coat color are still elusive in goats, and GWAS involving a large number of individuals as well as molecular studies are necessary to identify plausible candidate mutations.

### Selected genes related to other traits in Chengdu Brown and Jintang Black goats

In this work, we also identified several genes under selection that may underlie important traits in goats. In CB, for example, *POU1F1* and *GHRH* may play a role in reproduction or growth traits, as shown in other goat breeds [104–106], sheep [107], and cattle [108, 109]. Although there have been few reports about its role in important traits in domestic animals [110, 111], *GABRA2* is also regarded as a strong signature of selection. This gene encodes a receptor for GABA that is a major inhibitory neurotransmitter and has therefore been related to anxiety [112], depression [113], and particularly alcohol dependence [114–116].

In JT, the *LCORL* gene has been assumed to be a strong selection signal and was previously detected in Egyptian goats, particularly in Nubian goats [7]. This genomic region was first identified as a major QTL affecting carcass weight in Japanese Black cattle [117], and many studies have subsequently shown that the *NCAPG*-*LCORL* region, which exerts a pleiotropic effect, is significantly associated with growth (e.g., feed intake), body size (e.g., body weight), and reproduction traits (e.g., direct calving ease) in livestock, particularly in cattle (for a comprehensive review, see Takasuga [118]).

## Conclusions

After domestication, the genetic composition of Chengdu Brown goats from the Sichuan Basin became highly distinctive due to geographic isolation. Our work highlights genes that very likely contributed to breed standard traits. Taken together, these results provide an improved understanding of the evolutionary history of the domestic goat genome and its genes that have undergone artificial selection or are involved in the adaptation to local environments.

## Supplementary information

**Additional file 1.** Additional materials and methods.

**Additional file 2: Table S1.** Summary of mapping statistics for the three Chinese goat breeds that were sequenced in this study.

**Additional file 3: Table S2.** Summary of mapping statistics for the downloaded goat genome sequence data.

**Additional file 4: Table S3.** Summary of the SNPs and indels detected in the nine goat populations analyzed in this study.

**Additional file 5: Table S4.** Summary of the functional annotation of SNPs and indels detected in the nine goat populations analyzed in this study.

**Additional file 6: Figure S1.** Summary of genome-wide π values and ROH in six goat populations (CB, JT, TC, MD, MN, and Bezoar ibex). (a) Genome-wide π values in 10-kb sliding windows in these six goat populations. (b) Genomic patterns of homozygosity in these six goat populations. The total length of the genome covered by ROH and the total number of ROH are plotted on the x- and y- axes, respectively. (c) The proportions of ROH numbers with different ROH sizes (0–250 kb, 250–500 kb, 500–1000 kb, and > 1000 kb) in these six goat populations.

**Additional file 7: Table S5.** Summary of ROH detected in six goat populations (CB, JT, TC, MD, MN, and Bezoar ibex).

**Additional file 8: Figure S2.** Maximum-likelihood tree and optimal number of migration events in the nine goat populations analyzed. (a) Maximum-likelihood tree without migration edges based on TreeMix. (b) and (c) 99.8% of the variance between the populations could be explained when m = 2.

**Additional file 9: Figure S3.** Genome-wide selection signals identified in Chengdu Brown and Jintang Black goats. (a) Distribution of log2(θπ ratios) and FST values calculated in 10-kb sliding windows between CB and Bezoar ibex. The data points in red (corresponding to the top 5% of the empirical distribution) indicate genomic regions under selection in CB. (b) Distribution of log2(θπ ratios) and FST values calculated in 10-kb sliding windows between JT and Bezoar ibex. The data points in red (corresponding to the top 5% of the empirical distribution) indicate genomic regions under selection in JT. (c) Manhattan plot of iHH12 across all autosomes plotted with different colors for CB. The iHH12 values were calculated in 10-kb sliding windows. (d) Manhattan plot of iHH12 across all autosomes with different colors for JT. The iHH12 values were calculated in 10-kb sliding windows. (e) A Venn diagram of the shared positively selected genes among the three Chinese goat populations (CB, JT, and TC).

**Additional file 10: Table S6.** Summary of the regions of positive selective sweep identified in three Chinese goat breeds (CB, JT, and TC)

**Additional file 11: Table S7.** Summary of the positively selected genes identified in three Chinese goat breeds (CB, JT, and TC).

**Additional file 12: Table S8.** Significantly enriched biological terms for the positively selected genes identified in three Chinese goat breeds (CB, JT, and TC)

**Additional file 13: Table S9.** windows identified in the comparison between Tibetan Cashmere goats and four lowland domestic goat breeds (CB, JT, MD, and MN).

**Additional file 14: Table S10.** Summary of the selected genes identified in the comparison between Tibetan Cashmere goats and four lowland domestic goat breeds (CB, JT, MD, and MN).

**Additional file 15: Table S11.** Significantly enriched terms for the selected genes identified in the comparison between Tibetan Cashmere goats and four lowland domestic goat breeds (CB, JT, MD, and MN).

**Additional file 16: Table S12.** Summary of SNVs in four examples of selection signals detected in Tibetan Cashmere and Jintang Black goats.

**Additional file 17: Figure S4.** Nucleotide diversity in 10-kb sliding windows for the selection signals surrounding KITLG in six goat populations (CB, JT, TC, MD, MN and Bezoar ibex).

## Data Availability

The raw genome sequence data of 38 sampled goats in this study are available from the NCBI SRA database (Accession Number(s) PRJNA548681).
