# Supplementary material for: Comparative genome analyses reveal the unique genetic composition and selection signals underlying the phenotypic characteristics of three Chinese domestic goat breeds
Source: Genet Sel Evol. 2019 Nov 26;51:70. doi: 10.1186/s12711-019-0512-4 (PMC6880376; doi:10.1186/s12711-019-0512-4)
Supplement: Supplementary file 1 — Additional file 1. Additional materials and methods. [file 12711_2019_512_MOESM1_ESM.docx]

**Additional Materials and Methods**

**Alignment and variant calling**

After the removal of read pairs containing adapter sequences, quality control of the raw reads was conducted using Trimmomatic [1] (v0.36), with the following parameters: LEADING: 20, TRAILING: 20, SLIDINGWINDOW: 4:20 and MINLEN: 50. The meanings of these parameters were provided in Table 1. The high-quality reads were then mapped against the goat reference genome [2] (assembly ARS1, https://asia.ensembl.org/index.htm) using the ‘mem’ algorithm of BWA [3] (v0.7.12) with the default parameters. Picard software (v2.10.6) (<http://broadinstitute.github.io/picard/>) was applied to remove duplicated reads, which was followed by local realignment around existing indels and base quality score recalibration using GATK [4] (v3.8-0).

**Table S1 Summary of the parameters used in Trimmomatic**

| **Parameters** | **Definitions** |
| --- | --- |
| LEADING | Cut bases off the start of a read, if below a threshold quality. |
| TRAILING | Cut bases off the end of a read, if below a threshold quality. |
| SLIDINGWINDOW | Performs a sliding window trimming approach. It starts scanning at the 5’ end and clips the read once the average quality within the window falls below a threshold. |
| MINLEN | Drop the read if it is below a specified length. |

To obtain quality variants, filtration of the raw variant calls (SNPs and indels) was performed using GATK with the following cut-offs: QUAL < 100.0, QD < 2.0, MQ < 40.0, FS > 60.0, SOR > 3.0, MQRankSum < -12.5, and ReadPosRankSum < -8.0 (Table 2). The high-confidence variant sites were then obtained after discarding the variants with a minor allele frequency (MAF) < 0.05 and > 10% missing genotypes at the meta-population/population level using VCFtools [5]. The biallelic SNPs were finally extracted and used for the subsequent analyses. In addition, SnpEff [6] (v4.3) was used for SNP variant annotation and effect prediction.

**Table S2 Summary of the parameters used in filtration of the raw variant calls**

| **Parameters** | **Definitions** |
| --- | --- |
| QUAL | A quality (Phred score) of a variant. |
| QD | Variant call confidence normalized by depth of sample reads supporting a variant. |
| MQ | Root Mean Square of the mapping quality of reads across all samples. |
| FS | Strand bias estimated using Fisher's Exact Test. |
| SOR | Strand bias estimated by the Symmetric Odds Ratio test. |
| MQRankSum | Rank Sum Test for mapping qualities of REF versus ALT reads. |
| ReadPosRankSum | Rank Sum Test for relative positioning of REF versus ALT alleles within reads. |

**Additional Reference:**

1. Bolger AM, Lohse M, Usadel B. Trimmomatic: a flexible trimmer for Illumina sequence data. Bioinformatics. 2014; 30:2114-20.

2. Bickhart DM, Rosen BD, Koren S, Sayre BL, Hastie AR, Chan S, et al. Single-molecule sequencing and chromatin conformation capture enable de novo reference assembly of the domestic goat genome. Nat Genet. 2017; 49:643.

3. Li H, Durbin R. Fast and accurate short read alignment with Burrows–Wheeler transform. Bioinformatics. 2009; 25:1754-60.

4. Mckenna A, Hanna ME, Sivachenko A, Cibulskis K, Kernytsky A, Garimella K, et al. The Genome Analysis Toolkit: a MapReduce framework for analyzing next-generation DNA sequencing data. Genome Res. 2010; 20:1297-303.

5. Danecek P, Auton A, Abecasis G, Albers CA, Banks E, DePristo MA, et al. The variant call format and VCFtools. Bioinformatics. 2011; 27:2156-8.

6. Cingolani P, Platts A, Wang LL, Coon M, Nguyen T, Wang L, et al. A program for annotating and predicting the effects of single nucleotide polymorphisms, SnpEff. Fly. 2012; 6:80-92.
