# Supplementary figures and images for: Comparative genome analyses reveal the unique genetic composition and selection signals underlying the phenotypic characteristics of three Chinese domestic goat breeds
Source: Genet Sel Evol. 2019 Nov 26;51:70. doi: 10.1186/s12711-019-0512-4 (PMC6880376; doi:10.1186/s12711-019-0512-4)

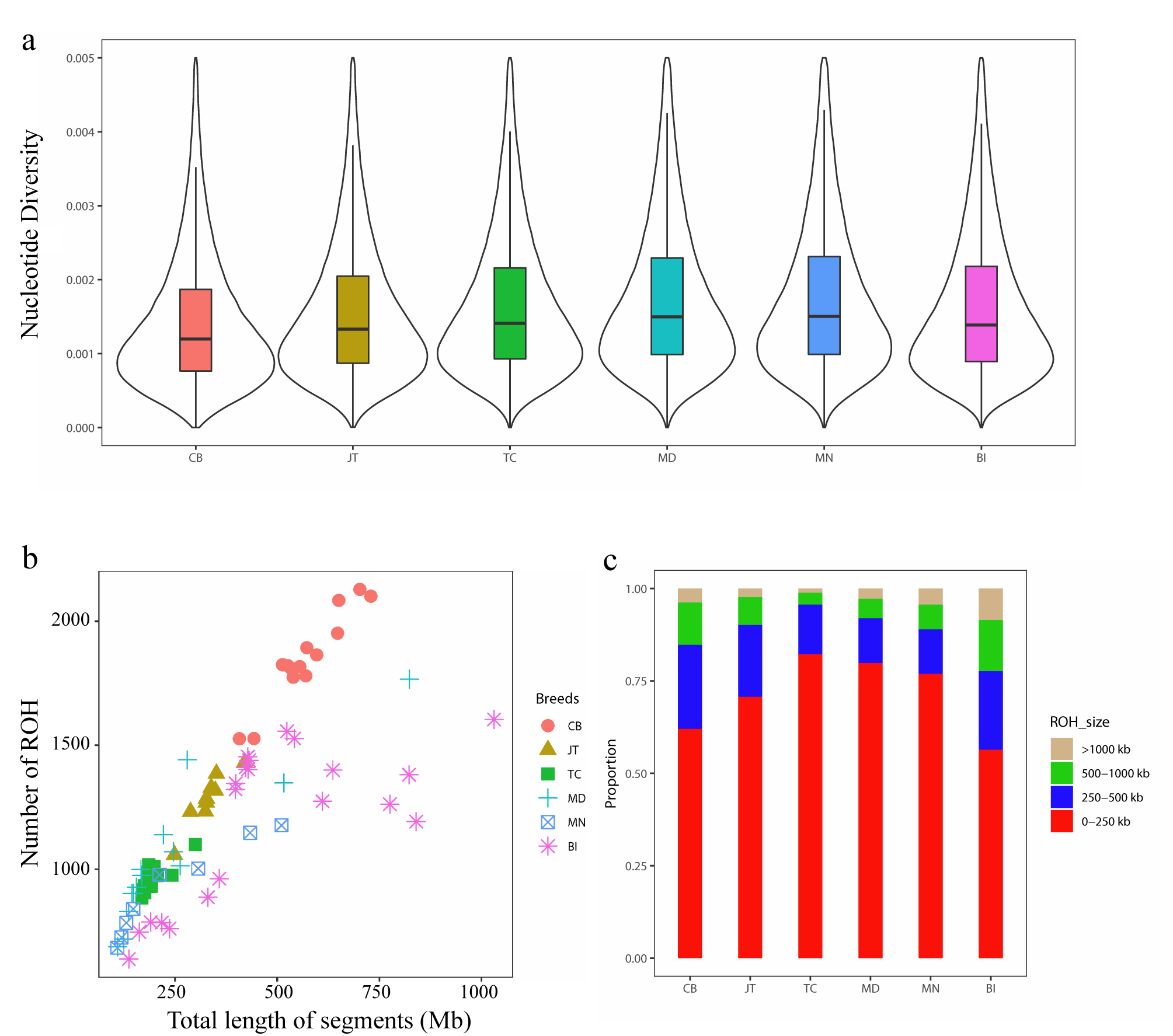

Supplement: Supplementary file 6 — Additional file 6: Figure S1. Summary of genome-wide π values and ROH in six goat populations (CB, JT, TC, MD, MN, and Bezoar ibex). (a) Genome-wide π values in 10-kb sliding windows in these six goat populations. (b) Genomic patterns of homozygosity in these six goat populations. The total length of the genome covered by ROH and the total number of ROH are plotted on the x- and y- axes, respectively. (c) The proportions of ROH numbers with different ROH sizes (0–250 kb, 250–500 kb, 500–1000 kb, and > 1000 kb) in these six goat populations. [file 12711_2019_512_MOESM6_ESM.tif]

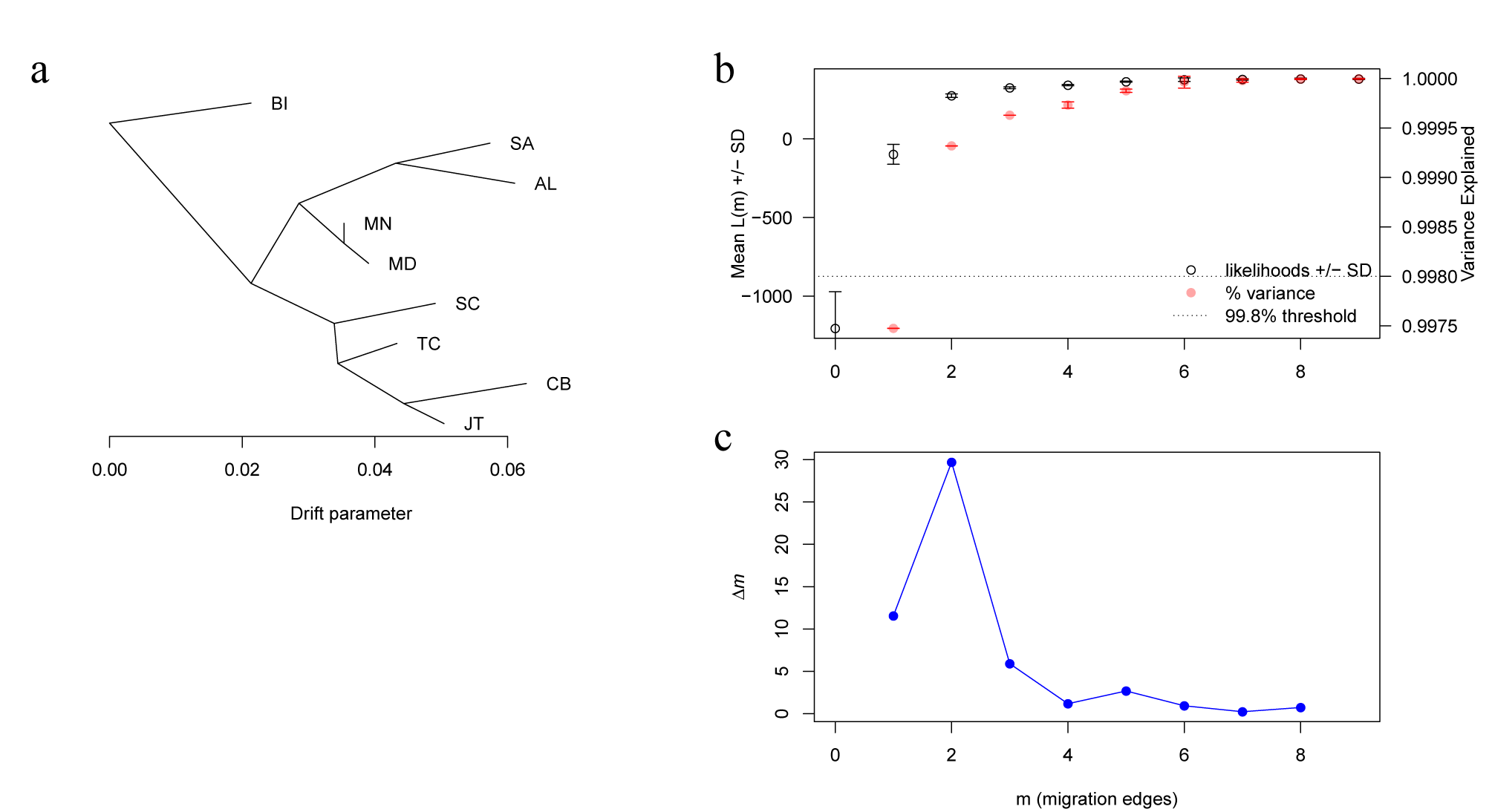

Supplement: Supplementary file 8 — Additional file 8: Figure S2. Maximum-likelihood tree and optimal number of migration events in the nine goat populations analyzed. (a) Maximum-likelihood tree without migration edges based on TreeMix. (b) and (c) 99.8% of the variance between the populations could be explained when m = 2. [file 12711_2019_512_MOESM8_ESM.tif]

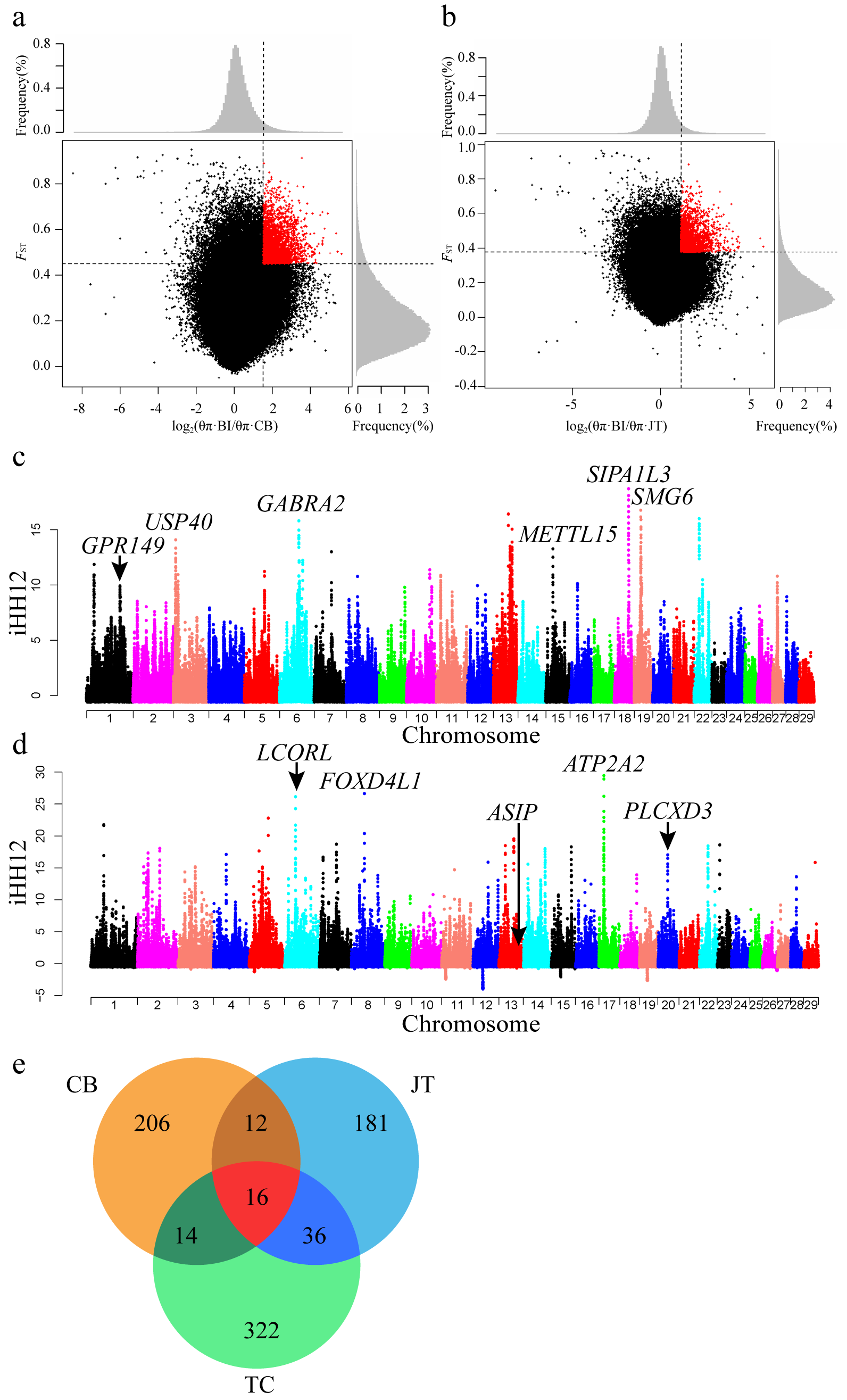

Supplement: Supplementary file 9 — Additional file 9: Figure S3. Genome-wide selection signals identified in Chengdu Brown and Jintang Black goats. (a) Distribution of log2(θπ ratios) and FST values calculated in 10-kb sliding windows between CB and Bezoar ibex. The data points in red (corresponding to the top 5% of the empirical distribution) indicate genomic regions under selection in CB. (b) Distribution of log2(θπ ratios) and FST values calculated in 10-kb sliding windows between JT and Bezoar ibex. The data points in red (corresponding to the top 5% of the empirical distribution) indicate genomic regions under selection in JT. (c) Manhattan plot of iHH12 across all autosomes plotted with different colors for CB. The iHH12 values were calculated in 10-kb sliding windows. (d) Manhattan plot of iHH12 across all autosomes with different colors for JT. The iHH12 values were calculated in 10-kb sliding windows. (e) A Venn diagram of the shared positively selected genes among the three Chinese goat populations (CB, JT, and TC). [file 12711_2019_512_MOESM9_ESM.tif]

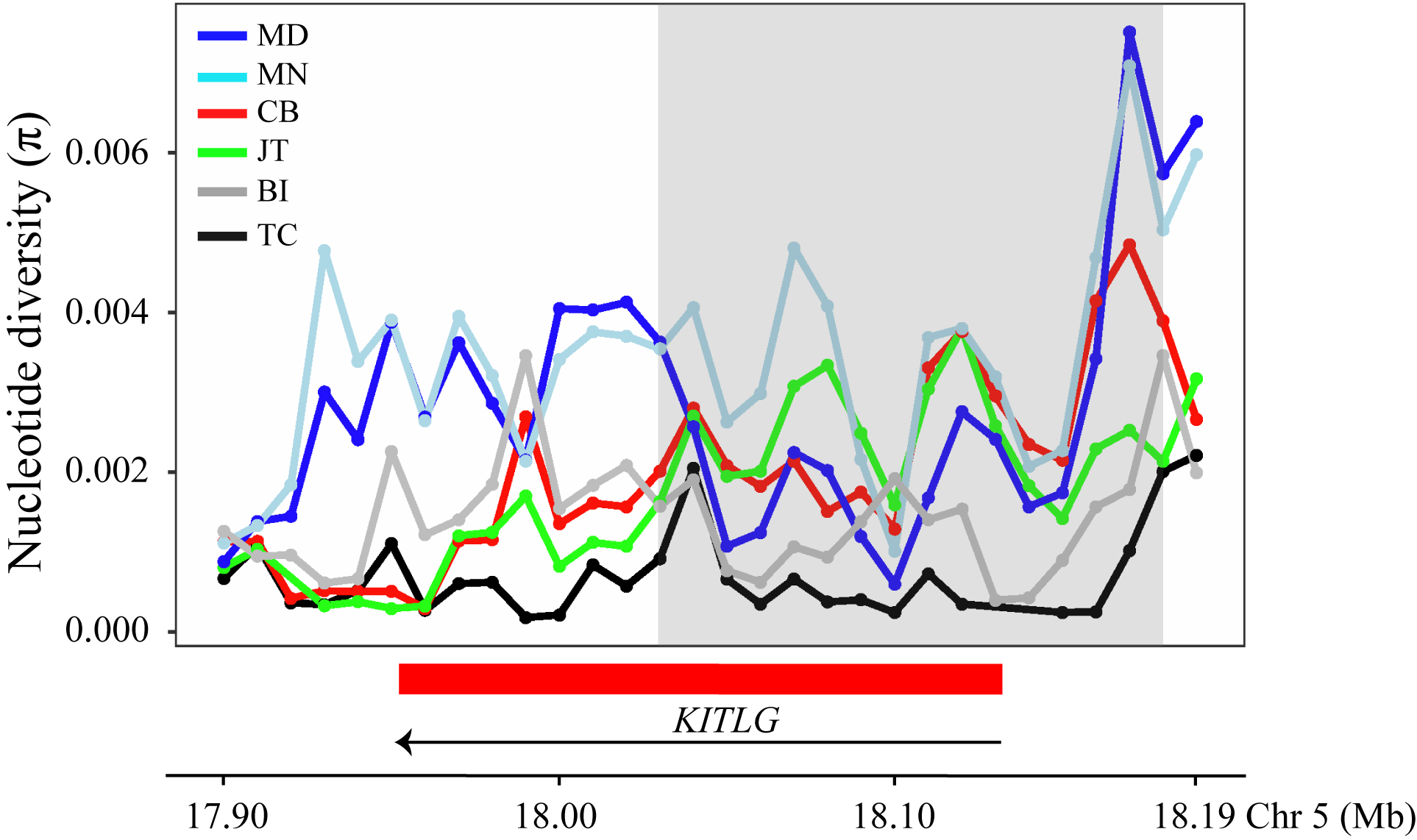

Supplement: Supplementary file 17 — Additional file 17: Figure S4. Nucleotide diversity in 10-kb sliding windows for the selection signals surrounding KITLG in six goat populations (CB, JT, TC, MD, MN and Bezoar ibex). [file 12711_2019_512_MOESM17_ESM.tif]
